# Diabetic Retinopathy Assessment Variability Among Eye Care Providers in an Urban Teleophthalmology Program

**DOI:** 10.1089/tmj.2018.0019

**Published:** 2019-04-10

**Authors:** Yao Liu, Victoria P. Rajamanickam, Ravi S. Parikh, Stephanie J. Loomis, Carolyn E. Kloek, Leo A. Kim, Dorothy L. Hitchmoth, Brian J. Song, Dean C. Xerras, Louis R. Pasquale

**Affiliations:** ^1^ Department of Ophthalmology and Visual Sciences, University of Wisconsin School of Medicine and Public Health, Madison, Wisconsin.; ^2^ Department of Biostatistics and Medical Informatics, University of Wisconsin School of Medicine and Public Health, Madison, Wisconsin.; ^3^ Department of Ophthalmology and Visual Science, Yale School of Medicine, New Haven, Connecticut.; ^4^ Massachusetts Eye and Ear Infirmary, Harvard Medical School, Boston, Massachusetts.; ^5^ White River Junction Veteran's Affairs Medical Center, Veteran's Health Administration, White River Junction, Vermont.; ^6^ Harvard Medical School, Massachusetts General Hospital Chelsea HealthCare Center, Boston, Massachusetts.; ^7^ Channing Division of Network Medicine, Brigham and Women's Hospital, Boston, Massachusetts.

**Keywords:** *telemedicine*, *ophthalmology*, *teleophthalmology*, *e-Health*

## Abstract

***Background:*** *Teleophthalmology is an evidence-based method for diabetic eye screening. It is unclear whether the type of eye care provider performing teleophthalmology interpretation produces significant variability.*

***Introduction:*** *We assessed grading variability between an optometrist, general ophthalmologist, and retinal specialist using images from an urban, diabetic retinopathy teleophthalmology program.*

***Methods:*** *Three readers evaluated digital retinal images in 100 cases (178 eyes from 90 patients with type 2 diabetes). Fisher's exact test, percent agreement, and the observed proportion of positive (P_pos_) or negative agreement (P_neg_) were used to assess variability.*

***Results:*** *Among cases deemed gradable by all three readers (n = 65), there was substantial agreement on absence of any retinopathy (88% ± 4.6%, P_neg_ = 0.91–0.95), presence of moderate nonproliferative or worse retinopathy (87% ± 3.9%, P_pos_ = 0.67–1.00), and presence of macular edema (99% ± 0.9%, P_pos_ = 0.67–1.00). There was limited agreement regarding presence of referable nondiabetic eye pathology (61% ± 11%, P_pos_ = 0.21–0.59) and early, nonroutine referral for a follow-up clinical eye exam (66% ± 8.1%, P_pos_ = 0.19–0.54). Among all cases (n = 100), there was acceptable agreement regarding which had gradable images (77% ± 5.0%, P_pos_ = 0.50–0.90).*

***Discussion:*** *Inclusion of multiple types of eye care providers as teleophthalmology readers is unlikely to produce significant variability in the assessment of diabetic retinopathy among high-quality images. Greater variability was found regarding image gradability, nondiabetic eye pathology, and recommended clinical referral times.*

***Conclusions:*** *Our results suggest that more extensive training and uniform referral standards are needed to improve consensus on image gradability, referable nondiabetic eye pathology, and recommended clinical referral times.*

## Introduction

An estimated 29.1 million American adults have diabetes mellitus and among them, approximately one quarter have diabetic retinopathy.^1^ Early detection and treatment dramatically reduce the risk of severe vision loss, yet diabetic retinopathy remains the leading cause of blindness among working-age U.S. adults, largely due to low screening rates.^2^ However, fewer than 50% of Americans with diabetes mellitus receive yearly diabetic eye screening as recommended by the American Diabetes Association (ADA), the American Academy of Ophthalmology (AAO), and the American Optometric Association (AOA).^2–4^

An urgent need exists for improved access to diabetic eye screening. The rapidly increasing prevalence of diabetes—expected to more than double by the year 2050—has created a growing demand for screening that cannot be met by the current eye care provider workforce.^5,6^ As an alternative to traditional screening methods, where an eye care provider performs a dilated eye examination, teleophthalmology programs using store-and-forward retinal photographs have been well validated to provide high-quality diabetic eye screening, to improve access to care, and to reduce blindness.^7–10^ Teleophthalmology is an evidence-based, cost-effective approach to diabetic eye screening that represents one of many dramatic advances in telemedicine on the verge of significant widespread adoption in the United States.^11,12^ These retinal photographs can be obtained in the primary care setting where 90% of patients with known diabetes obtain medical care.^13^ This technology is particularly well suited for preventing blindness in urban minority populations such as Latinos, who are at greater risk for vision loss due to a higher prevalence of more severe diabetic retinopathy and lower screening rates.^14^

While teleophthalmology has become increasingly accepted for diabetic eye screening in primary care settings, the availability of adequately trained, qualified readers may limit its widespread implementation.^15,16^ Prior studies have compared many different types of readers, including general practitioners, nurses, optometrists, and ophthalmologists.^15^ Most U.S. teleophthalmology programs currently use eye care providers as readers, but training backgrounds among these readers can vary significantly. It remains unclear as to whether the type of eye care provider performing teleophthalmology interpretation produces significant variability. This study is the first to directly assess variability between an optometrist, a general ophthalmologist, and a fellowship-trained retina specialist for teleophthalmology evaluation of diabetic retinopathy.

## Methods

### Teleophthalmology Image Acquisition and Presentation

Three readers assessed a set of masked telemedicine cases from 90 patients (178 eyes) with type 2 diabetes mellitus, who participated in a clinical teleophthalmology program for diabetic eye screening. All images were acquired by a trained ophthalmic photographer from 2008 to 2010 at a single urban primary care clinic affiliated with Massachusetts General Hospital located in Chelsea, Massachusetts, using a nonmydriatic camera (Topcon TRC NW-6S, Topcon Medical Systems, Inc., Tokyo, Japan). Four nonmydriatic, nonstereoscopic digital images were acquired from each eye following the validated Joslin 3-field technique (i.e., 45-degree views of the posterior pole, area nasal to the disc, and area superotemporal to the disc) plus an external photograph.^17^ Images from both eyes were included with the exception of 2 cases with monocular patients in whom their only eye was imaged. To assess intrareader reliability, 10 cases were duplicated and all 3 readers independently assessed 100 masked case vignettes from 90 patients. In addition to retinal photographs, each masked telemedicine case included a brief synopsis of the patient's demographic information and medical history, including age, gender, self-reported ethnicity, diabetes type, diabetes medications, last hemoglobin A1c, and past medical history. Readers viewed all masked telemedicine cases within Microsoft PowerPoint (Microsoft, Inc., Redmond, WA).

### Reader Backgrounds and Grading Instructions

The readers were a Veteran's Affairs (VA) Medical Center optometrist (D.H.), an academic general ophthalmologist (C.K.), and an academic fellowship-trained retina specialist (L.K.). All readers completed the standard teleophthalmology diabetic retinopathy reader training program (an electronic independent study course) instituted by the VA healthcare system before initiation of the study. All were active readers in clinical teleophthalmology programs, but the optometrist had several years more of experience from her prior participation in the VA teleophthalmology program.

Readers had not previously evaluated any of the teleophthalmology images in this study and did not have access to the patients' prior clinical assessments for diabetic retinopathy. All readers were oriented to the navigation of the telemedicine case vignettes and the electronic grading sheet by the same investigator (Y.L.) using a series of five sample cases that were not included in the data analysis. Readers were instructed on how to adjust images as needed to maximize gradability, including adjustment for brightness and contrast, as well as for increasing magnification over any area of interest. Readers were informed that the intent of the study was to assess variability in telemedicine diabetic retinopathy assessments.

The readers were asked to determine whether or not images from each case were of sufficient quality to be gradable for diabetic retinopathy, whether there was any diabetic retinopathy present, the severity of diabetic retinopathy if present, and whether there was referable nondiabetic eye pathology (i.e., glaucomatous optic nerve, advanced macular degeneration, etc.). For each case, readers selected one of the three following options for recommended referral time to a clinical eye examination: (1) urgent/within 1 month, (2) nonurgent/within 2–6 months, or (3) routine/within 1 year. Diabetic retinopathy was evaluated on a three-level severity scale: (1) none, (2) mild nonproliferative, or (3) moderate nonproliferative or worse based on the International Classification of Diabetic Retinopathy.^18^ If images were considered ungradable, then all remaining assessments regarding the absence or presence of retinopathy, macular edema, or nondiabetic eye pathology were categorized as “unable to determine.” There were no synchronous, clinical dilated eye examinations or dilated seven-field ETDRS retinal photographs to serve as reference standards because study images were previously acquired in an active clinical teleophthalmology program.^19^

### Statistical Analysis

Comparisons of inter-reader and intrareader variability were assessed using Fisher's exact test and percent agreement. In addition, a statistical measure known as the observed proportion of positive (P_pos_) or negative agreement (P_neg_) was used in place of kappa (κ) to confirm agreement due to the “kappa paradox,” wherein kappa values are artificially low due to the infrequency of retinopathy in this community-based sample.^20,21^ Values of 70–90% agreement are generally considered acceptable for inter-reader reliability.^22,23^ Percent agreement has been used in similar studies and can be safely used since readers were well-trained eye care providers who were unlikely to be guessing in their assessments.^8,24^ Agreement was considered limited if <70%, acceptable if 70% or greater, and substantial if 80% or greater. All statistical calculations were made using Stata software (StataCorp LP, College Station, TX).

### Ethics/IRB Approval

This study was approved by the Human Studies Committee/Institutional Review Board at the Massachusetts Eye and Ear Infirmary and complied with the tenets of the Declaration of Helsinki.

## Results

Masked telemedicine cases in this study included patients (*n* = 90) who were predominantly Latino (73.4%) and female (60%) with an average age of 54 years (range: 28–83 years) (*Table 1*). All had a diagnosis of type 2 diabetes mellitus with an average hemoglobin A1c of 7.5% (range: 5.2–12.1%).

**Table 1.** Patient Demographics (*n* = 90)

Age	54 years (range: 28–83 years, median 52.5 years)
Female	54 (60%)
Ethnicity, %	
Latino/Hispanic	73.4
Caucasian, non-Latino/Hispanic	14.4
African American	8.9
Middle Eastern	2.2
Asian	1.1
Diagnosis of type 2 diabetes mellitus	100%
Average hemoglobin A1c	7.5% (range: 5.2–12.1), 58 mmol/mol (range: 33–109)

Among all three readers in this study, the average percentage of all telemedicine cases (*n* = 100) found to have clinically significant diabetic retinopathy (i.e., moderate nonproliferative or worse) was 3.7% ± 1.5% (range: 2.0–5.0%) and the average percentage of cases found to have macular edema was 2.7% ± 0.6% (range 2.0–3.0%) (*Table 2*). These values were not significantly different (*p* > 0.05) among the three readers. The frequency of cases with images considered ungradable for diabetic retinopathy was variable—27% by the general ophthalmologist, 17% by the optometrist, and 3% by the retina specialist (*p* < 0.005). However, inter-reader agreement regarding gradable images for diabetic retinopathy among all cases (*n* = 100) was acceptable (77% ± 5.0%, P_pos_ = 0.50–0.90).

**Table 2.** Image Gradability, Diabetic Retinopathy Severity, and Macular Edema in All Cases (*n* = 100)

	OPTOMETRIST, %	GENERAL OPHTHALMOLOGIST, %	RETINA SPECIALIST, %	AVERAGE ± STANDARD DEVIATION, %	p^a^
Ungradable images	17	27	3.0	16 ± 12	**<0.005**
No retinopathy	70	56	89	72 ± 17	**<0.005**
Any retinopathy	13	17	8.0	12 ± 4.5	0.17
Mild retinopathy	11	12	4.0	9.0 ± 4.4	0.09
Moderate or worse retinopathy	2.0	5.0	4.0	3.7 ± 1.5	0.64
Macular edema	2.0	3.0	3.0	2.7 ± 0.6	1.0

^a^ Fisher's exact test.

To assess inter-reader variability in diabetic retinopathy evaluation, we examined the subset of teleophthalmology cases (*n* = 65) with high-quality images considered gradable for retinopathy by all three readers (*Table 3*). In this subset, there was substantial agreement on: (1) the absence of any retinopathy (88% ± 4.6%, P_neg_ = 0.91–0.95), (2) the presence of moderate nonproliferative or worse retinopathy (i.e., severity of retinopathy) (87% ± 3.9%, P_pos_ = 0.67–1.00), and (3) the presence of macular edema (99% ± 0.9%, P_pos_ = 0.67–1.00). Of note, all cases (*n* = 7) found to have clinically significant diabetic eye disease (i.e., macular edema and/or moderate nonproliferative or worse retinopathy) by the retina specialist were also identified as such by the other two readers. Referral recommendations were compared as follows: agreement on early, nonroutine (i.e., urgent or nonurgent) referral (P_pos_) and agreement on routine referral (P_neg_). There was limited inter-reader agreement regarding the presence of referable nondiabetic eye pathology (61% ± 11%, P_pos_ = 0.21–0.59) and earlier, nonroutine referral for a follow-up clinical eye examination (66% ± 8.1%, P_pos_ = 0.19–0.54).

**Table 3.** Inter-Reader Agreement in Cases Gradable for Diabetic Retinopathy by All Readers (*n* = 65)

	OPTOMETRIST VS. GENERAL OPHTHALMOLOGIST		OPTOMETRIST VS. RETINAL SPECIALIST		GENERAL OPHTHALMOLOGIST VS. RETINA SPECIALIST		AVERAGE ± STANDARD DEVIATION
	PERCENT AGREEMENT	P_pos_ P_neg_	PERCENT AGREEMENT	P_pos_ P_neg_	PERCENT AGREEMENT	P_pos_ P_neg_	PERCENT AGREEMENT
Any retinopathy	92	P_pos_ = 0.76 P_neg_ = 0.95	88	P_pos_ = 0.20 P_neg_ = 0.93	83	P_pos_ = 0.15 P_neg_ = 0.91	88 ± 4.6
Severity of retinopathy	91	P_pos_ = 0.67 P_neg_ = 0.99	88	P_pos_ = 1.00 P_neg_ = 1.00	83	P_pos_ = 0.67 P_neg_ = 0.99	87 ± 3.9
Macular edema	99	P_pos_ = 0.67 P_neg_ = 0.99	99	P_pos_ = 0.67 P_neg_ = 0.99	100	P_pos_ = 1.00 P_neg_ = 1.00	99 ± 0.9
Referable nondiabetic eye disease	63	P_pos_ = 0.59 P_neg_ = 0.67	51	P_pos_ = 0.21 P_neg_ = 0.66	68	P_pos_ = 0.22 P_neg_ = 0.80	61 ± 11
Recommended referral time	51	P_pos_ = 0.54 P_neg_ = 0.62	45	P_pos_ = 0.19 P_neg_ = 0.60	74	P_pos_ = 0.33 P_neg_ = 0.85	66 ± 8.1

Intrareader agreement was more variable in each of the diagnostic and disposition categories (*Table 4*). Overall, there was greater intrareader agreement (80–90%, P_pos_ = 0.67–1.00) for the optometrist and retina specialist than for the general ophthalmologist (50–70%, P_pos_ = 0.60–0.77) on gradability of images for diabetic retinopathy, severity of retinopathy, and the presence of macular edema. The optometrist and general ophthalmologist had lesser intrareader agreement (60%, P_pos_ = 0.40–0.71) than the retina specialist (90%, P_pos_ = 0.86–1.00) regarding the presence of referable nondiabetic eye disease and earlier, nonroutine referral to a clinical examination.

**Table 4.** Intrareader Agreement in Duplicate Cases (*n* = 10)

	OPTOMETRIST		GENERAL OPHTHALMOLOGIST		RETINA SPECIALIST		AVERAGE ± STANDARD DEVIATION
	PERCENT AGREEMENT	P_pos_ P_neg_	PERCENT AGREEMENT	P_pos_ P_neg_	PERCENT AGREEMENT	P_pos_ P_neg_	PERCENT AGREEMENT
Gradable images	90	P_pos_ = 0.93 P_neg_ = 0.80	60	P_pos_ = 0.60 P_neg_ = 0.60	80	P_pos_ = 0.89 P_neg_ = 0.00	77 ± 15
Any retinopathy	90	P_pos_ = 1.00 P_neg_ = 1.00	60	P_pos_ = 0.77 P_neg_ = 0.57	90	P_pos_ = 0.80 P_neg_ = 0.93	80 ± 17
Severity of retinopathy	80	P_pos_ = 1.00 P_neg_ = 1.00	50	P_pos_ = 0.67 P_neg_ = 0.94	90	P_pos_ = 1.00 P_neg_ = 1.00	73 ± 21
Macular edema	90	P_pos_ = 0.86 P_neg_ = 0.92	70	P_pos_ = 0.73 P_neg_ = 0.67	90	P_pos_ = 0.67 P_neg_ = 0.94	83 ± 12
Referable nondiabetic eye disease	60	P_pos_ = 0.67 P_neg_ = 0.50	60	P_pos_ = 0.40 P_neg_ = 0.80	90	P_pos_ = 0.86 P_neg_ = 0.92	70 ± 17
Recommended referral time	60	P_pos_ = 0.71 P_neg_ = 0.33	60	P_pos_ = 0.60 P_neg_ = 0.60	90	P_pos_ = 1.00 P_neg_ = 1.00	70 ± 17

## Discussion

Our study demonstrated substantial inter-reader agreement in diabetic retinopathy assessment among the subset of teleophthalmology cases (*n* = 65) with high-quality images considered gradable by all three readers: an optometrist, a general ophthalmologist, and a fellowship-trained retina specialist. Prior studies have assessed variability among other types of teleophthalmology readers for diabetic retinopathy. Ruamviboonsuk et al. evaluated inter-reader agreement between three retina specialists, three general ophthalmologists, three ophthalmic nurses, and three ophthalmic photographers in single-field digital retinal photographs.^15^ Notably, their study did not include optometrists, who form the majority of readers in the VA healthcare system—one of the largest U.S. teleophthalmology programs.^11^ A 2-day instruction course was provided to all nonphysician readers in that study, but no standardized training was provided to general ophthalmologists or retina specialists. The authors found only fair agreement between and within groups, except for retina specialists who showed moderate to substantial intragroup agreement. Vujosevic et al. demonstrated similar findings among retina specialists.^25^ Longer training may be needed for nonphysician readers, who showed moderate to substantial agreement with retina specialists after 1 year of rigorous training in a study by Bhargava et al.^16^ While using nonphysician readers may expand reader availability, investment in a year-long training program may not be practical in many U.S. healthcare settings. In this study, all readers were eye care providers who underwent a brief electronic independent self-study training program instituted by the VA healthcare system.

Ruamviboonsuk et al. concluded that retinal specialists were generally the most reliable teleophthalmology readers.^15^ It is therefore notable that in our study, all cases identified by the retina specialist as having clinically significant diabetic eye disease (i.e., those with macular edema or moderate nonproliferative or worse retinopathy) were also identified as such by the other two readers. Therefore, if we were to use the retina specialist's interpretation as a reference standard, then the general ophthalmologist and optometrist both had 100% sensitivity in identifying vision-threatening diabetic eye disease for early clinical referral, although they referred additional patients (e.g., 70% and 81% specificity, respectively) often as a result of noting more ungradable images.^18^ A previous study comparing the results of in-person clinical diabetic eye examinations between optometrists and general ophthalmologists found that optometrists had a referral sensitivity of 77% and 92% for moderate to severe macular edema and diabetic retinopathy, respectively.^26^ Our study demonstrates that telemedicine evaluation by non-retina specialist, eye care providers is highly sensitive for detecting clinically significant, vision-threatening forms of diabetic eye disease when a standardized training program is used and furthermore, that this appears to exceed clinical thresholds currently accepted for in-person dilated eye examinations.

Since most patients in our study did not have retinopathy, it was unsurprising that there was greater agreement on the absence of any retinopathy (P_neg_) versus its presence (P_pos_) and greater agreement on absent or mild retinopathy (P_neg_, severity of retinopathy) versus moderate or worse retinopathy (P_pos_, severity of retinopathy) due to the difference in the frequencies of these findings in the community. Similarly, because of the lower frequency of referable nondiabetic eye disease and earlier, nonroutine referral, there was greater agreement on the absence of referable nondiabetic eye disease (P_neg_) versus its presence (P_pos_) and greater agreement on routine (P_neg_, recommended referral time) versus earlier, nonroutine referral (P_pos_, recommended referral time).

In addition to inter-reader agreement, we also evaluated intrareader agreement by duplicating 10 cases in our dataset. The general ophthalmologist had the lowest intrareader agreement, in part, from having a high percentage (27%) of ungradable image assessments, which led to more frequent assessments of “unable to determine” in each category. The higher frequency of ungradable image assessments by the general ophthalmologist may reflect less experience compared to the retina specialist in evaluating diabetic retinopathy under suboptimal conditions (e.g., through a postoperative gas bubble). Nor did the general ophthalmologist have as much experience with teleophthalmology as the VA optometrist. These data support the existence of a learning curve in teleophthalmology grading among eye care providers.^15^ As a result, teleophthalmology programs should consider having more experienced readers supervise newer readers with protocols for on-going quality assurance, wherein more than one reader assesses a subset of images (“over-reads”).

The large range of ungradable rates (3–27%) found among our readers is similar to that of other published studies (4–35%).^8,17,27^ Since the absence of retinopathy is more common in our clinical image dataset, the fact that the optometrist and general ophthalmologist noted more ungradable images than did the retina specialist also resulted in their noting fewer cases with no diabetic retinopathy. The wide range of gradability found by readers in our study suggests that improved training modules to standardize criteria for gradability should be developed. This is especially important because unnecessarily high ungradable rates could strain downstream eye care services (e.g., patients with ungradable images are typically referred for clinical evaluation because 50% have significant ocular pathology).^28^ In addition, there was limited inter-reader agreement on referable nondiabetic eye disease and recommended clinical referral times. Owsley et al. have found that 44.2% of patients participating in teleophthalmology screening for diabetic retinopathy have nondiabetic eye pathology.^29^ Given the high frequency of nondiabetic eye pathology in teleophthalmology programs and the need to regulate the volume of downstream eye clinic referrals, training modules to further standardize referral criteria in these areas are also needed.

One proposed solution to inter-reader variability is automated grading using objective software algorithms. Prior studies have shown that automated grading systems have high sensitivity, but low specificity.^30^ A more recent study by Gulshan et al. showed both high sensitivity and specificity for detecting moderate or worse diabetic retinopathy using a deep learning algorithm.^31^ However, costs for licensing such software, professional liability, and billing models remain to be determined. Also, these algorithms may be limited in their ability to correctly assess the relative significance of a wide variety of nondiabetic eye pathology.^32^ Thus, automated grading systems may be best used in combination with human readers. Future studies should evaluate the optimal implementation of automated grading systems in clinical teleophthalmology programs.

A major strength of our study is that the telemedicine cases came directly from a clinical teleophthalmology program for diabetic retinopathy screening in an urban, primary care clinic. In addition, our study population was unique given the large proportion (73.4%) of Latino patients. This group has been found to have an increased prevalence, incidence, and likelihood of progression of diabetic retinopathy as well as lower rates of diabetic eye screening compared to other ethnic groups.^14,33^ Latinos and other high-risk groups with historically low screening rates have the greatest potential to benefit from increased access to diabetic eye screening using teleophthalmology. Thus, it is important for these groups to be well represented in teleophthalmology research studies. Interestingly, the frequency of diabetic retinopathy was substantially lower in our study than that reported previously in a Los Angeles Latino population.^34^ This may have resulted, in part, from a lack of patients with type 1 diabetes in our study. In addition, patients with type 2 diabetes obtaining regular care at this community-based primary care clinic may have relatively better long-term glycemic control than those in other parts of the country with less access to care.

Limitations of our study included the absence of a reference standard for the telemedicine cases (e.g., synchronous in-person examination or ETDRS 7-field images) since our cases came directly from a real-world teleophthalmology program.^19^ Furthermore, a single individual represented each type of eye care provider in our study. Some of our results may be attributable to unique aspects of each reader and larger studies with more readers may help to determine the generalizability of our findings. Although all readers underwent standardized training, there were no interim assessments to provide readers with additional feedback. Such feedback may be critical for ongoing quality assurance, and the grading of a subset of reads by at least one other experienced reader should be used for quality control purposes in teleophthalmology programs. Another limitation was the small number of duplicates (10%) used to assess intrareader variability. However, this exceeded the proportion of duplicate reads (5%) used as quality control standards for retinal image assessments in large-scale research studies.^35^

In summary, substantial inter-reader agreement was found among three telemedicine readers: an optometrist, general ophthalmologist, and retinal specialist in diabetic retinopathy assessment among cases with high-quality images. This is the first study directly comparing variability in diabetic teleophthalmology evaluation between readers with these training backgrounds. Our study suggests that the inclusion of multiple types of eye care providers as teleophthalmology readers is unlikely to produce significant variability in diabetic retinopathy grading when a standardized training program is used. Training modules to further standardize criteria for image gradability, referable nondiabetic eye pathology, and recommended clinical referral times should be developed. Continued advances in image assessment are needed to expand access to diabetic eye screening using teleophthalmology to meet the growing demand in communities worldwide.
